# Investigating Ammonium By-product Removal for Ureolytic Bio-cementation Using Meter-scale Experiments

**DOI:** 10.1038/s41598-019-54666-1

**Published:** 2019-12-04

**Authors:** Minyong Lee, Michael G. Gomez, Alexandra C. M. San Pablo, Colin M. Kolbus, Charles M. R. Graddy, Jason T. DeJong, Douglas C. Nelson

**Affiliations:** 1 Department of Civil and Environmental Engineering, University of Washington, Seattle, WA 98195 USA; 2 Department of Civil and Environmental Engineering, University of California, Davis, CA 95616 USA; 3 Department of Microbiology and Molecular Genetics, University of California, Davis, CA 95616 USA

**Keywords:** Civil engineering, Applied microbiology, Environmental chemistry

## Abstract

Microbially Induced Calcite Precipitation (MICP), or bio-cementation, is a promising bio-mediated technology that can improve the engineering properties of soils through the precipitation of calcium carbonate. Despite significant advances in the technology, concerns regarding the fate of produced NH_4_^+^ by-products have remained largely unaddressed. In this study, five 3.7-meter long soil columns each containing one of three different soils were improved using ureolytic bio-cementation, and post-treatment NH_4_^+^ by-product removal was investigated during the application of 525 L of a high pH and high ionic strength rinse solution. During rinsing, reductions in aqueous NH_4_^+^ were observed in all columns from initial concentrations between ≈100 mM to 500 mM to final values between ≈0.3 mM and 20 mM with higher NH_4_^+^ concentrations observed at distances furthest from the injection well. In addition, soil V_s_ measurements completed during rinse injections suggested that no significant changes in cementation integrity occurred during NH_4_^+^ removal. After rinsing and a 12 hour stop flow period, all column solutions achieved cumulative NH_4_^+^ removals exceeding 97.9%. Soil samples collected following rinsing, however, contained significant sorbed NH_4_^+^ masses that appeared to have a near linear relationship with surrounding aqueous NH_4_^+^ concentrations. While these results suggest that NH_4_^+^ can be successfully removed from bio-cemented soils, acceptable limits for NH_4_^+^ aqueous concentrations and sorbed NH_4_^+^ masses will likely be governed by site-specific requirements and may require further investigation and refinement of the developed techniques.

## Introduction

Microbially Induced Calcite Precipitation (MICP), or bio-cementation, has shown significant promise as an environmentally-conscious alternative to geotechnical ground improvement technologies, which have traditionally relied upon hazardous grouting chemicals, high mechanical energy, and energy-intensive materials to improve the engineering properties of soils^1–3^. In the urea hydrolysis (ureolysis) driven process, microorganisms containing urease enzymes are used to catalyze a reaction that degrades urea, yielding total ammonium (NH_4_^+^), dissolved inorganic carbon, and hydroxide ions^4^ [Eqs. (1–3)]. When soluble calcium is available from either treatment solutions or groundwater, resulting alkalinity can supersaturate solutions with respect to calcium carbonate (CaCO_3_) and initiate mineral precipitation on soil particle surfaces and contacts [Eq. (4)]. Resulting bio-cementation can improve the engineering properties of soils through large increases in shear stiffness and strength with small reductions in hydraulic conductivity and porosity^5–8^. The process has been proposed for a variety of applications including mitigation of earthquake-induced soil liquefaction, general geotechnical soil improvement, subsurface flow manipulation, and divalent contaminant immobilization among other uses^1,9–12^.

1 $${({N}{{H}}_{2})}_{2}{CO}+2{{H}}_{2}{O}\to 2{N}{{H}}_{3}+{H}_{2}C{O}_{3}$$

2 $$N{H}_{3}+{H}_{2}O\leftrightarrow N{H}_{4}^{+}+O{H}^{-}$$

3 $${H}_{2}C{O}_{3}\leftrightarrow HC{O}_{3}^{-}+{H}^{+}\leftrightarrow C{O}_{3}^{-2}+2{H}^{+}$$

4 $$C{a}^{+2}+C{O}_{3}^{-2}\leftrightarrow CaC{O}_{3(solid)}$$

Researchers have made significant advances in the technology in recent years, including identifying alternative treatment techniques^13,14^, up-scaling the process to meter-scale^15–19^, and characterizing the engineering behavior of bio-cemented soils^8,20^. Despite these advances, environmental concerns regarding the fate of produced NH_4_^+^ by-products have remained largely unaddressed^1,21^. NH_4_^+^ is a commonly encountered source of inorganic nitrogen in soil systems, however, high aqueous NH_4_^+^ concentrations produced following ureolytic MICP may present serious environmental and human health concerns if left untreated in soils and groundwater. For example, the presence of high NH_4_^+^ in surface waters can encourage the growth of toxic algal blooms, which can decrease aquatic dissolved oxygen availability, produce high levels of toxins, and encourage bacterial growth, therefore threatening fish, humans, and other flora and fauna^22^. While no maximum contaminant level has been established by the U.S. EPA for total NH_4_^+^ in drinking water, maximum concentrations for aquatic life of 17 mg/L (≈1 mM) and 1.9 mg/L (≈0.1 mM) total NH_4_^+^ for acute and chronic exposure, respectively, have been recommended^23^. During field-scale applications, aqueous NH_4_^+^ concentrations produced during bio-cementation will likely require removal to meet water quality standards and or maintain similar water quality as that present prior to treatments. NH_4_^+^ concentrations near twice the concentration of applied urea are expected by reaction stoichiometry [Eq. (1)], with many past experiments resulting in the production of NH_4_^+^ concentrations exceeding 500 mM. Limited understanding of post-treatment NH_4_^+^ removal has been a significant barrier for bio-cementation technology that has limited field-scale applications and environmental benefits. In order for MICP to be a truly environmentally beneficial technology, methods to manage, remediate, or remove NH_4_^+^ by-products following bio-cementation are needed.

While *in-situ* nitrification of produced NH_4_^+^ to nitrite (NO_2_^−^) and nitrate (NO_3_^−^) (under aerobic conditions) and subsequent denitrification of NO_3_^−^ to nitrogen gases (under anaerobic conditions) may offer a potential remediation strategy, these processes will be challenging and likely unpractical at field-scale due to the need for dramatic modifications in subsurface oxygen availability and chemical conditions. In addition, Gat *et al*.^21^ showed that *in-situ* oxidation of NH_4_^+^ can have detrimental effects on bio-cementation integrity resulting from the generation of acidity in this process, further suggesting the need to address produced NH_4_^+^ following bio-cementation. Although few studies have examined and quantified the removal of NH_4_^+^ by-products following MICP, “rinse” solutions have been most commonly applied to remove NH_4_^+^ from treated areas with subsequent remediation of collected effluent completed *ex-situ* at water reclamation facilities^15,24^^.^ Post-treatment rinsing may require significant energy and materials when applied at field scale, however, the technique can provide an effective strategy for NH_4_^+^ management following bio-cementation in the absence of other remediation processes and may provide an opportunity for NH_4_^+^ recovery. Recently, centimeter-scale soil column experiments were performed to further investigate and quantify the removal of NH_4_^+^ by-products following MICP in a clean poorly-graded Concrete Sand material using rinse solution injections^25^. Results suggested that removal of positively-charged NH_4_^+^ ions from clean sands may be significantly more difficult due to interactions with negatively-charged soil particle surfaces. When rinse solutions of differing ionic strength and pH were applied, NH_4_^+^ removal was improved with increases in ionic strength, while pH had no detectable influence on removal. Improved removal was attributed to the exchange of NH_4_^+^ existing on soil surfaces with Ca^2+^ cations supplied from rinse solutions. A high pH (pH = 9.0) and high ionic strength (500 mM CaCl_2_) rinse solution was identified that improved NH_4_^+^ removal while simultaneously limiting dissolution of bio-cementation. Although these results were promising, it remained unclear if these techniques would remain effective at meter-scale treatment distances.

In this study, five 3.7-meter long soil columns were improved using ureolytic bio-cementation, and post-treatment NH_4_^+^ by-product removal was investigated as a function of different soil materials and treatment techniques. Columns contained three different poorly-graded sand materials including: an alluvial sand (Column 1, 2, and 3), a marine sand (Column 4), and a second alluvial sand material (Column 5). Prior to bio-cementation, all columns received different biological treatments over the first six days intended to either enrich native ureolytic microorganisms or augment *Sporosarcina pasteurii (S. pasteurii)* bacteria uniformly across column lengths. Three different biological treatment strategies were applied to enrich native ureolytic microorganisms to achieve high (Column 1) and low bulk ureolytic rates (Column 2, 4, 5) and augment soils with *S. pasteurii* at a high cell density (Column 3) to obtain a high ureolytic rate similar to Column 1. Differences in achieved ureolytic rates were intended to examine the effect of urea hydrolysis rate on the spatial uniformity and extent of bio-cementation; the results of which are more extensively described in a separate manuscript^26^. Following biological treatments, nine cementation injections containing identical calcium and urea concentrations were applied to all columns over nine (Column 1, 3) and eighteen (Column 2, 4, 5) days. All columns achieved differing degrees of bio-cementation with distance from the injection well. Following MICP treatments, 525 L of a high pH and high ionic strength rinse solution was applied to each column to remove produced NH_4_^+^. During rinse injections, changes in NH_4_^+^ by-product removal and effects on bio-cementation integrity were monitored. Following rinsing, physical soil samples were collected at various locations and sorbed NH_4_^+^ masses remaining on soil surfaces were quantified.

## Materials and Methods

### Soil materials

Four different clean poorly-graded sands were used to prepare soil columns. Column 1, 2, and 3 contained the same alluvial Concrete Sand used in past experiments^18,25,27,28^, Column 4 contained a marine Delta Sand, and Column 5 contained an alluvial Covelo Sand. In all columns, coarser Monterey Sand was placed at column ends as a filter material. Soil properties including USCS classification following ASTM D2487-10^29^, depositional environment, D_10_, D_30_, D_60_, fines content (% <#200 sieve), and minimum (e_min_) and maximum (e_max_) void ratios are summarized for all sands in Table 1.

**Table 1:** Summary of Soil Properties.

Soil Material	USCS	Deposition	D_10_ (mm)	D_30_ (mm)	D_60_ (mm)	Fines Content (%)	e_min_	e_max_
Concrete Sand	SP	Alluvial	0.23	0.54	1.54	1.1	0.35	0.60
Delta Sand	SP	Marine	0.19	0.25	0.37	1.3	0.48	0.95
Covelo Sand	SP	Alluvial	0.23	0.52	1.55	1.6	0.38	0.59
Monterey Sand	SP	Marine	1.01	1.15	1.45	0	—	—

### Soil columns

**Figure 1:**
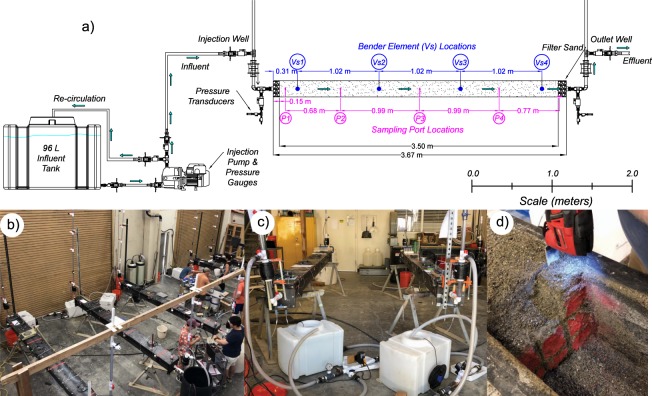
Overview of meter-scale experiments including: (**a**) schematic of meter-scale soil columns with treatment application systems, sampling ports, and bender element locations, (**b**) images of soil columns during treatments, (**c**) treatment solution injection systems, and **(d**) bio-cemented soil materials during post-rinsing soil sampling.

Five 3.7-meter long hollow steel columns with square cross-sections (0.2 m by 0.2 m) contained 0.15 m^3^ of sand and received treatment injections in one direction to simulate a single stream tube within a well-to-well half-space. Treatment wells (ID = 26.6 mm) were located on column ends and had valves, which allowed for pressurization of the injection well and removal of effluent solutions at the outlet well. Soils were placed in three ≈16 to 76 mm lifts, which allowed for bender element sensors to be placed at mid-height within columns during soil placement. Lifts were moist tamped and surfaces were scarified to limit density differences between lifts. Table 2 summarizes soil material types, soil column porosities, relative densities, and pore volumes determined from placed soil masses. In all columns, estimated porosities were between 0.30 and 0.40, relative densities were between 55% and 67%, and column pore volumes (PV) varied between 48.5 L and 63.0 L. Columns contained four bender element sensor pairs at distances of 0.31 m, 1.33 m, 2.35 m, and 3.37 meters from the injection location to monitor changes in soil shear wave velocities (V_s_), indicative of bio-cementation. Four aqueous sampling ports were placed at distances of 0.15 m, 0.83 m, 1.82 m, and 2.81 m away from the injection location to monitor injection pressures and obtain solution samples at various times. Sampling ports consisted of 0.15 m long steel tubes (ID = 3.35 mm) with 25.4 mm diameter circular plastic filters (125–195 μm) open at mid-height within columns. A fifth drain port, consisting of a 60.3 mm ball valve with a porous filter material, was used to obtain additional samples and was located 3.58 m away from the injection location at the bottom of columns. Figure 1 provides a detailed schematic and images of soil columns including treatment application systems, bender element and sampling port locations, and treatment wells.

**Table 2:** Summary of Soil Column Properties and PHREEQC Model Parameters.

Column No.	Experimental Measurements				PHREEQC Model Parameters	
	Soil Material	Porosity	Relative Density (%)	Total Pore Volume (L)	Porosity	Longitudinal Dispersivity (m)
1	Concrete Sand	0.31	56	50.7	0.23	0.3
2	Concrete Sand	0.30	67	48.5	0.23	0.3
3	Concrete Sand	0.32	56	50.6	0.25	1.5
4	Delta Sand	0.40	58	63.0	0.33	0.1
5	Covelo Sand	0.32	55	50.3	0.25	1.5

### Saturation and bromide passive tracer testing

Prior to all treatments, columns were slowly saturated with an artificial ground water (AGW) solution containing 40 μM KNO_3_, 450 μM MgSO_4_, 1.75 mM CaCl_2_, 40 μM NaNO_3_, 1.1 mM NaHCO_3_, and 60 μM KHCO_3_ following Ferris *et al*.^30^. Immediately following saturation and before all bio-cementation treatments, columns received passive tracer injections to evaluate differences in solution transport between columns. During tracer testing, 76 L of a 15 mM NaBr solution was injected, followed by 76 L of de-ionized water to examine the arrival and removal of passive Br^−^ ions at the outlet well. A constant flow rate of 400 mL/min was used for injections and solution samples were collected at outlet wells once every 5 minutes. Solution conductivities were measured and normalized by the conductivity of the injected NaBr solution to estimate normalized Br^-^ concentrations (C/C_0_). One-dimensional advective-dispersive soil column transport models were developed using the geochemical software PHREEQC^31^ and were used to match passive tracer experimental observations by varying porosities and longitudinal dispersivities for all columns. All models were composed of 42 cells (0.089 meters each) and received NaBr and de-ionized water injections that were identical to the physical experiment.

### Treatment injections

Following passive tracer testing, all columns received different treatment solutions in a series of three treatment phases: (1) enrichment/augmentation, (2) cementation, and (3) NH_4_^+^ rinsing. Table 3 presents a summary of treatment schemes including solution chemical constituents and concentrations, injection numbers and volumes, and injection and stop flow time durations for all columns. In the first treatment phase, solutions were applied to either enrich soil columns for native ureolytic microorganisms (Column 1, 2, 4, 5) or augment soils with the highly active ureolytic bacterium*, S. pasteurii* (Column 3). Enriched columns received six enrichment treatments once daily with varying concentrations of yeast extract intended to achieve different ureolytic activities. Column 1 received higher yeast extract concentrations (0.2 g/L) following past experiments^14^ wherein high ureolytic activities were targeted (hydrolysis of 250 mM urea within ≈8 hours). Columns 2, 4, and 5 received lower yeast extract concentrations (0.04 g/L) to obtain a low bulk ureolytic rate (hydrolysis of 250 mM urea within ≈48 hours). Column 3 was inoculated on the last day of enrichment treatments by injecting 76 L of an isotonic saline solution (9 g/L NaCl) containing *S. pasteurii* (ATCC strain 11859) at a cell density of 9.36 × 10^7^ cells/mL, intended to match the high ureolytic rate of Column 1. Due to lower ureolytic activity than expected along Column 3, a second augmentation injection consisting of 456 L of isotonic saline with 1.4 × 10^6^ cells/mL was applied after the fourth cementation injection. Following all enrichment treatments, a flush solution that was identical to cementation solutions, but did not contain urea and Ca^2+^, was applied to all enriched columns immediately before the first cementation treatment to remove high aqueous carbonate species expected after enrichment. Cementation treatments containing Ca^2+^ were then applied to all columns to initiate CaCO_3_ precipitation for a total of nine injections. Columns 1 and 3 received cementation treatments once every 24 hours and Columns 2, 4, and 5 received treatments once every 48 hours to allow near full hydrolysis to occur. During enrichment, augmentation, and cementation treatments, injection volumes of 76 L were applied at a constant flow rate of 400 mL/min, during which injected solutions were continuously mixed within injection tanks. Following cementation treatments, a 200 mM CaCl_2_ solution (initial pH ≈ 10.0) was applied to all columns to remove NH_4_^+^ following previously identified strategies^25^. During NH_4_^+^ by-product removal injections, a rinse solution volume of 525 L was injected into each column at a flow rate of 750 mL/min and lasted ≈700 minutes. Following rinse injections, columns remained saturated for 12 hours during a stop flow period until columns were disassembled. All solutions were injected using small pumps (Wayne Inc., 0.1 HP) with pressure gauges to monitor injection pressures.

**Table 3:** Summary of Solution Constituents and Injection Schemes for All Columns.

Solution Constituent	Column 1		Column 2, 4, & 5		Column 3		All Columns
	Enrichment	Cementation	Enrichment	Cementation	Augmentation	Cementation	NH_4_^+^ Rinsing
Calcium Chloride (mM)	—	250	—	250	—	250	200
Urea (mM)	50	250	50	250	—	250	—
Ammonium Chloride (mM)	100	12.5	100	12.5	—	12.5	—
Sodium Acetate (mM)	42.5	42.5	42.5	42.5	—	42.5	—
Yeast Extract (g/L)	0.2	0.2	0.04	0.02	—	—	—
Sodium Hydroxide (g/L)	1.28	—	1.28	—	—	—	—
NaCl (g/L)	—	—	—	—	9	—	—
S. pasteurii (cells/mL)	—	—	—	—	9.36 × 10^7^* 1.40 × 10^6^**	—	—
Initial Solution pH	9.0	8.4	9.0	8.4	≈7.0	8.4	10.0
Number of Injections	6	9	6	9	2	9	1
Injection Volume (L)	76	76	76	76	76* 456**	76	525
Injection Duration (min)	186	186	186	186	186* 1,140**	186	700
Stop Flow Period (hours)	20.8	20.8	20.8	44.8	24.5	20.8	12.3

*First augmentation treatment.

**Second augmentation treatment.

### Aqueous sampling

Before and immediately after treatment injections, aqueous samples of ≈10 mL were collected from all sampling ports. On select days, aqueous samples were also collected 1, 2, 4, and 8 hours after injections to monitor ureolytic activity and chemical changes in time. Prior to all sample collection events, 30 mL of solution was removed from ports and discarded to obtain representative samples. During NH_4_^+^ rinse injections, ≈10 mL samples were collected from outlet wells once every 20 minutes and from all sampling ports and well locations once every 60 minutes. Aqueous samples were immediately frozen and stored at −20 °C until subsequent chemical analyses.

### Shear wave velocity measurements

Shear wave velocity (V_s_) measurements were obtained using horizontally-oriented bender element sensor pairs placed at mid-depth within columns at various distances from the injection well. V_s_ measurements were obtained at all locations before and immediately after all biological and cementation injections. Additional measurements were performed during NH_4_^+^ rinsing before, 120, 240, 480, and 700 minutes after the start of rinse injections and following a 12-hour stop flow period (24 hours after start of rinse injections). Bender elements were coated with epoxy, electronics wax, and an insulating coating to waterproof sensors following similar processes detailed in Gomez *et al*.^18^. Transmitting bender elements were excited with a 24 V 100 Hz square wave and signals from receiving bender elements were measured and recorded using an oscilloscope at a sampling frequency of 1 MHz. Shear wave arrival times were interpreted visually and V_s_ values were calculated from measured sensor spacings.

### Aqueous measurements

Solution pH measurements were completed using a semi-micro pH electrode and meter system that was calibrated daily using a three-point buffer sequence (4.01, 7.00, 10.00) and had ± 0.01 pH unit accuracy. Total NH_4_^+^ measurements were completed using a salicylate reaction method similar to Krom (1980)^32^, wherein two reagents (Reagent A & B) were added to dilute sample volumes and absorbance values were measured at 650 nm using a microplate spectrophotometer. Reagent A consisted of 1.9 mM sodium nitroprusside, 811 mM sodium salicylate, 387 mM sodium citrate, and 515 mM sodium tartrate in water. Reagent B consisted of 1.32 mM sodium hypochlorite and 1.5 M sodium hydroxide in water. Urea measurements were completed using a colorimetric urea assay similar to Knorst *et al*.^33^. A colorimetric reagent consisting of 216 mM p-dimethylaminobenzaldehyde, 2.32 M hydrochloric acid, and 13.83 M ethanol in water was added to dilute samples and absorbance values were measured at 422 nm using a microplate spectrophotometer. Conductivity measurements were completed using a conductivity probe and meter. Augmented cell densities were determined through direct counting^34^.

### Cation exchange capacity and exchangeable cation measurements

Cation exchange capacity (CEC) and exchangeable cation measurements were completed for untreated sand materials using a process similar to U.S. EPA Method 9080^35^. Soil CEC values reflect the capacity of negatively-charged soil surfaces to bind positively-charged ions, thus it was hypothesized that CEC differences between soils could influence NH_4_^+^ transport and removal following bio-cementation. During these measurements, 10 grams of untreated dry soil and 50 mL of a 1 M NH_4_Cl solution were added to a plastic syringe. After a 12-hour residence period, soil solutions were extracted, collected, and select exchangeable cations were characterized using inductively coupled plasma mass spectrometry (ICP-MS). Remaining extracted soil samples were then rinsed with ethanol for 6 hours to remove NH_4_^+^ ions that may have remained in free solution. Finally, 50 mL of 1 M KCl solution was added to all samples and allowed to equilibrate for 12 hours to encourage replacement of sorbed NH_4_^+^. Soil solutions were then extracted again and NH_4_^+^ concentrations in the extracted solution were quantified using the salicylate colorimetric assay. Measured NH_4_^+^ concentrations were used to calculate soil CEC values.

### Soil sampling and soil NH_4_^+^ measurements

Following all treatments, column top caps were removed, and soil samples were collected at various locations at the center of columns. At heavily cemented locations, soil samples required removal using an oscillating power saw (Fig. 1d). Following sample collection, moist soil samples were frozen and stored at −20 °C until subsequent chemical analyses could be completed. An extraction process was used to quantify NH_4_^+^ masses remaining within soil samples. Free soil solution was first removed from thawed moist soil samples using a centrifuge process wherein 30 grams of moist soil samples (of known water content) were added to conical centrifuge tubes with 0.45 micron nylon filter baskets and centrifuged at 4150 rpm for 20 minutes to extract solutions. A minimum volume of 2 mL was collected, frozen, and stored at −20 °C until NH_4_^+^ concentrations were analyzed. Sorbed NH_4_^+^ masses remaining on soil particle surfaces were quantified using a KCl extraction process following Keeney & Nelson (1982)^36^. In this process, 10 gram moist soil samples (of known water content) were mixed with 20 mL of a 2 M KCl solution and allowed to equilibrate for at least 4 hours to facilitate removal of NH_4_^+^ ions. Soil solution mixtures were then added to another conical filter tube, centrifuged, and a 2 mL solution sample was collected, frozen, and stored at −20 °C until NH_4_^+^ concentrations were analyzed. KCl extracted NH_4_^+^ measurements included NH_4_^+^ initially present in free soil solutions as well as NH_4_^+^ masses that were initially sorbed to soil surfaces. Sorbed NH_4_^+^ masses were therefore estimated by subtracting NH_4_^+^ masses expected from free solution from NH_4_^+^ measurements following KCl extraction. Sorbed NH_4_^+^ masses were normalized per gram of dry soil.

### Statement on consent to publish

The authors have notified persons shown in Fig. 1 and have received consent to publish images.

## Results and Discussion

Results from soil CEC and exchangeable cation measurements are summarized below in Table 4 for all three sands. A fourth sample consisting of Concrete Sand augmented with 10% by mass reagent-grade CaCO_3_ was also tested to assess the potential influence of CaCO_3_ minerals on soil CEC. As shown, Delta Sand had the highest CEC (4.32 meq/100 g) with Covelo Sand exhibiting the lowest CEC of tested sands (1.64 meq/100 g). Additionally, when CaCO_3_ was added to the Concrete Sand sample, no significant effects on CEC were observed suggesting that the presence of bio-cementation CaCO_3_ minerals likely had little influence on the ability of soils to bind NH_4_^+^ ions. When examining exchangeable cation concentrations, sands generally had similar values for common soil cations. Delta Sand, however, had notably higher K^+^, Mg^2+^, Na^+^, and S^2+^ concentrations than other tested sands, which was consistent with the marine depositional environment from which it was obtained. Exchangeable cations in Concrete Sand and Covelo Sand were most similar, however, Covelo Sand had significantly higher Al^3+^ and Ca^2+^ concentrations as well as much lower Mg^2+^ concentrations than other sands. While tested sands had minor CEC and exchangeable cation differences, values were generally consistent with those expected for clean quartz sands^37^.

**Table 4:** Summary of Cation Exchange Capacity and Exchangeable Cation Analyses for Sands.

Sand	CEC (meq per 100 g soil)	Exchangeable Cations (μg per gram of soil)									
		Al^3+^	Ba^2+^	Ca^2+^	K^+^	Mg^2+^	Mn^2+^	Na^+^	S^2+^	Si^4+^	Zn^2+^
Concrete Sand	2.58	<0.1	21.4	277.7	18.9	191.5	<0.1	23.9	3.6	9.9	<0.1
Delta Sand	4.32	<0.1	13.5	236.7	84.0	238.8	1.5	180.1	81.8	9.6	<0.1
Covelo Sand	1.64	0.7	9.4	555.3	18.7	59.6	1.3	5.7	17.0	4.8	2.3
Concrete Sand w/10% CaCO_3_	2.52	<0.1	14.8	910.1	16.7	129.5	<0.1	17.8	3.7	6.7	<0.1

**Figure 2:**
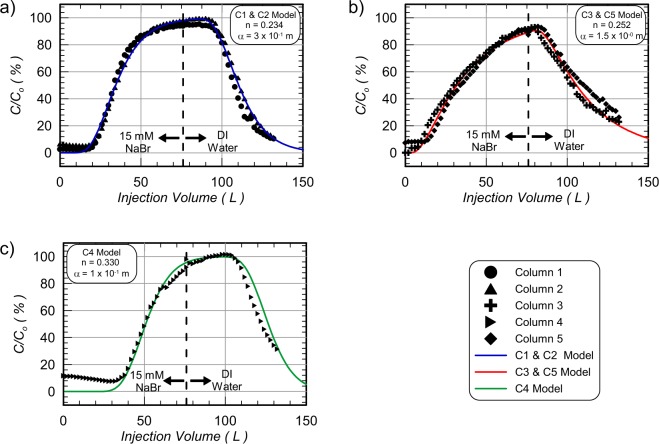
Measurements of normalized bromide concentrations (C/C_0_) versus injected volume during passive tracer testing for (**a**) Column 1 and 2, (**b**) Column 3 and 5, and (**c)** Column 4 with PHREEQC modelled comparisons.

Figure 2 presents measurements of normalized solution conductivity (C/C_0_) versus injected NaBr tracer solution volume from samples obtained at outlet wells during passive tracer testing. Differences in C/C_0_ values with injected volume are reflective of porous media advective-dispersive transport properties including porosity, hydrodynamic dispersion, and diffusion^38^. Immediately after starting the 15 mM NaBr solution injection, most C/C_0_ values were near zero with small values (<10% C/C_0_) observed exiting columns due to background soil solution conductivities. In Column 4, containing Delta Sand, notably larger initial C/C_0_ values were observed and were consistent with the higher exchangeable cations measured previously (Table 4). After additional tracer solution was injected, C/C_0_ values increased above background levels (C/C_0_ ≈ 20%) in Column 1, 2, 3, and 5 after injecting ≈20 to 30 L (0.39 to 0.62 PV). Column 4 had a much more delayed arrival, however, with C/C_0_ values increasing above ≈20% only after ≈42 L (0.66 PV). The delayed breakthrough in Column 4 was consistent with the higher porosity measured in this column (Table 2). After injecting the 76 L volume, C/C_0_ values measured at the outlet well were between 94% and 96% for Column 1 and 2, 89% and 90% for Column 3 and 5, and were 98% for Column 4. While it was expected that Column 1, 2, and 3, which all contained Concrete Sand, would have similar breakthrough curves, the Column 3 breakthrough was distinctly different. Column 3 and 5, however, were found to have very similar breakthrough curve behaviors despite containing different sands. While unexpected, both Concrete Sand and Covelo Sand had similar grain size distributions and differences in transport properties may have resulted from differences in soil preparation between columns and minor preferential flow at the column boundaries. To better quantify transport differences between columns one-dimensional advective-dispersive soil column transport models were calibrated to match experimental trends by varying column longitudinal dispersivities (α) and porosities (n). Modelled results are presented in Fig. 2 and porosity and dispersivity values used for models are summarized in Table 2. Modelled porosities were lower than those calculated from soil mass measurements for all columns, likely due to incomplete saturation, which could have reduced the apparent porosity of columns. When comparing dispersivity values between columns, a large increase in longitudinal dispersivities was observed between Column 1 and 2 (α = 0.3 m) and Column 3 and 5 (α = 1.5 m), despite having similar porosities. Column 4 had a similar dispersivity (α = 0.1 m) as Column 1 and 2, but a much larger modeled porosity (n_model_ = 0.33) as expected from soil mass measurements.

**Figure 3:**
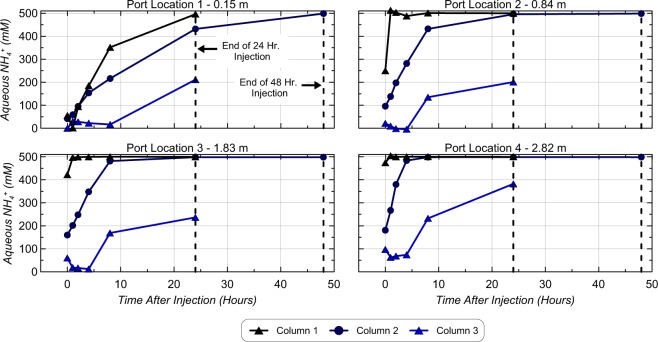
Changes in aqueous NH_4_ + concentrations in time at all aqueous sampling port locations during the ninth cementation treatment.

Figure 3 presents aqueous NH_4_^+^ concentrations in time following the ninth cementation injection for Column 1, 2, and 3 from measurements at all port locations, immediately after injections, and 1, 2, 4, 8, 24, and 48 hours (when applicable) after injections. Aqueous NH_4_^+^ concentrations were determined by difference and reaction stoichiometry from direct urea measurements in time. While similar data were also obtained for Column 4 and 5, only Column 2 data are shown and was representative of trends observed in all enriched low ureolysis rate columns (Column 2, 4, 5). As shown, similar NH_4_^+^ production trends in time were observed between columns at each port location. Immediately after injections, aqueous NH_4_^+^ concentrations were lowest at the 0.15 m port location and increased with distance from the injection well for all columns due to urea hydrolysis occurring during solution transport as well as mixing with previously reacted solutions. At a distance of 0.15 m in the high ureolysis rate Column 1, NH_4_^+^ concentrations increased from post-treatment values near ≈50 mM to ≈500 mM over 24 hours, while ≈500 mM concentrations were achieved at all other ports within 1 hour after injections. In the low ureolysis rate Column 2, NH_4_^+^ concentrations increased from post-treatment values near ≈50 mM to ≈500 mM at a distance of 0.15 m within 48 hours, and to ≈500 mM at all other ports between 6 and 24 hours after injections. In the augmented Column 3, a slower rate of urea hydrolysis was consistently observed at all port locations relative to enriched columns. This resulted in significantly lower NH_4_^+^ concentrations between ≈200 mM and ≈400 mM residing after 24 hour treatment periods and a significant fraction of injected urea remaining non-hydrolyzed due to low ureolytic activity. Localization of ureolytic activity near injection well locations has been previously observed in augmented experiments^39^ and likely resulted from the colloidal filtration of *S. pasteurii* bacterial cells during augmentation of Column 3^40^. Despite much lower NH_4_^+^ production observed in Column 3, all enriched columns (1, 2, 4, 5) achieved similar post-treatment NH_4_^+^ concentrations near ≈500 mM after 24 hour (Column 1) and 48 hour residence periods (Columns 2, 4, 5).

**Figure 4:**
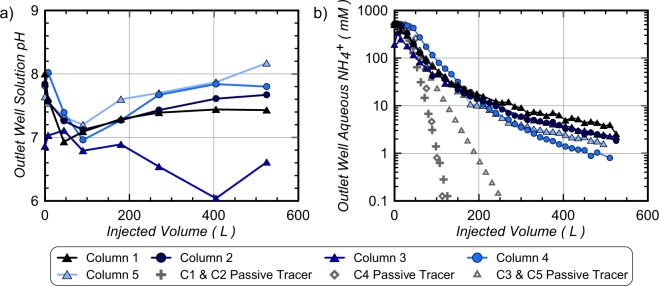
Measurements of outlet well (**a**) solution pH and (**b**) aqueous NH_4_^+^ concentrations versus injected rinse solution volume with PHREEQC modeled passive tracer comparisons.

Figure 4 presents solution pH and aqueous NH_4_^+^ concentration measurements for outlet well samples versus injected rinse solution volume. As shown in Fig. 4a, solutions initially exiting all enriched columns had pH values between 7.6 and 8.0. This was consistent with previously observed solution pH values for cementation solutions with urea-to-calcium ratios of 1:1 following near full urea hydrolysis. In Column 3, however, initial pH values were significantly lower (near 6.8) due to limited generation of alkalinity from limited urea hydrolysis. As rinse injections proceeded, effluent pH values approached 7.0 for all columns after injecting 100 L. In all enriched columns (Column 1, 2, 4, 5), pH values then gradually increased to near steady values between 7.4 and 8.2 for the remainder of rinsing. The temporary reduction observed in outlet solution pH values was unexpected as the initial pH of the injected rinse solution was near 10. It is hypothesized that this pH reduction may have resulted from consumption of remaining carbonate species and some limited calcite precipitation upon the initial introduction of rinse solutions with 200 mM Ca^2+^. Following the removal of sufficient solution alkalinity, increases in pH may then have occurred due to equilibration of high pH solutions with existing CaCO_3_ and soil minerals. In Column 3, pH values remained lower than all enriched columns with final outlet well solution pH values near 6 to 6.5, which likely resulted from a lack of significant quantities of CaCO_3_ minerals in this column.

Large reductions in effluent NH_4_^+^ from values between 497 mM and 524 mM (Column 1, 2, 4, 5) and ≈342 mM (Column 3) to values between ≈20 and 32 mM were observed in all columns at the outlet well after injecting 200 L (Fig. 4b). Following the application of an additional 200 L, however, NH_4_^+^ concentrations were only reduced to values between 3.5 and 9.0 mM. At the end of the 525 L injection, all effluent NH_4_^+^ concentrations were between 0.8 and 2.5 mM. It is hypothesized that the limited improvement in NH_4_^+^ removal after injecting significantly more rinse solution volume was influenced by soil-ion interactions and removal of sorbed NH_4_^+^ masses, though solution mixing via hydrodynamic dispersion may have also contributed. In order to evaluate the effect of these interactions on observed NH_4_^+^ trends, PHREEQC models were used to simulate passive tracer removal trends for all columns. As shown, concentrations were similar between measured NH_4_^+^ and modelled passive tracers early during rinsing due to limited breakthrough at the outlet well location, however, at injection volumes greater than 100 L, modeled passive tracer concentrations were significantly lower than experimentally observed NH_4_^+^ values. For example, reductions in passive ion concentrations to values below 10 mM required a maximum injection volume of 124 L for all models, however, physical experiments suggested that over twice that volume (≈280 L) was required to achieve these NH_4_^+^ levels. When integrating outlet well concentrations over injected volumes, measurements suggested the removal of 27.2, 27.2, 17.6, 38.6, and 28.2 moles of NH_4_^+^ occurred in Columns 1 through 5, respectively. This was equivalent to the removal of average pore fluid concentrations of 536 mM, 561 mM, 347 mM, 612 mM, and 560 mM NH_4_^+^ for Columns 1 through 5, respectively. Average pore fluid concentrations removed from Columns 1, 2, 4, and 5 exceeded the maximum expected NH_4_^+^ concentration of 500 mM and suggested that significant sorbed NH_4_^+^ masses must have been removed during rinsing. Retardation of NH_4_^+^ transport relative to passive ions was also previously observed in centimeter-scale experiments^25^ and likely contributed to delayed NH_4_^+^ removal relative to passive tracer trends. Lastly, although NH_4_^+^ removal was expected to be reduced in Column 4 for similar rinse injection volumes due to a larger porosity, surprisingly, when injection volumes exceeded ≈200 L, an opposite trend was observed with much lower NH_4_^+^ concentrations exiting Column 4 compared to other columns. While unexpected, this was consistent with the greater NH_4_^+^ removal calculated for this column.

**Figure 5:**
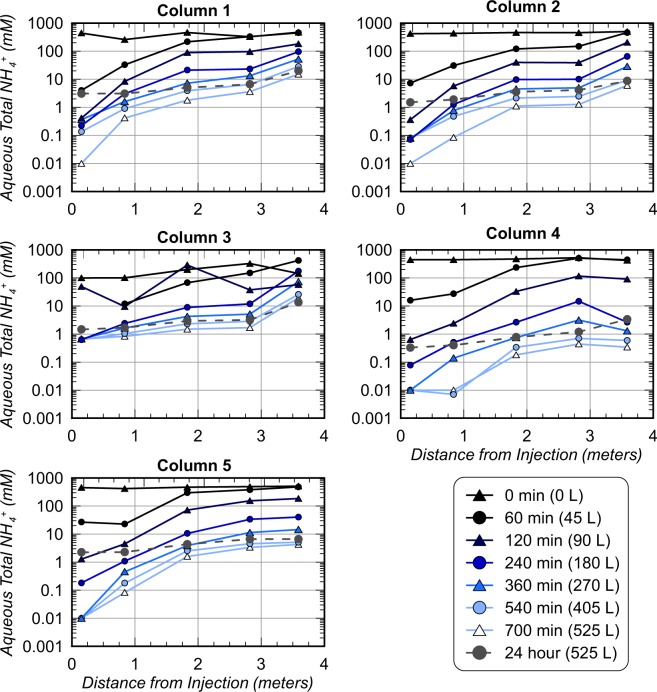
Contours of aqueous NH_4_^+^ concentrations within columns at all sampling port locations at various times during rinse injections. Measurement times after start of injections and cumulative injected rinse volumes are provided.

**Figure 6:**
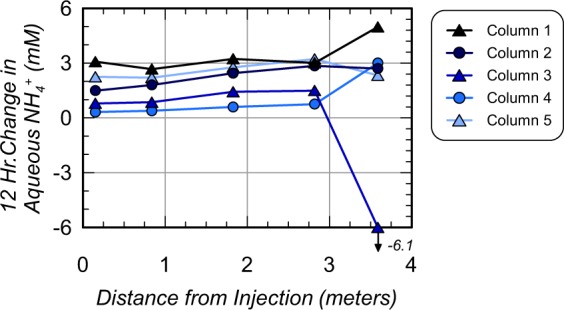
Changes in aqueous NH_4_^+^ concentrations following a 12 hour stop flow period after rinsing versus column length for all columns.

Figure 5 presents aqueous NH_4_^+^ concentrations measured spatially along soil columns at various points in time during rinse injections and 24 hours after rinse injections following a 12 hour stop flow period. Pre-rinsing measurements reflected conditions following the ninth cementation treatment, wherein most locations in enriched columns had NH_4_^+^ concentrations between ≈400 and 500 mM due to near full hydrolysis of the previously applied 250 mM urea injection. In Column 3, however, much lower NH_4_^+^ concentrations between 100 mM and 324 mM were observed and were consistent with the lower ureolytic activity observed in the augmented column during cementation treatments (Fig. 3). As rinse injections proceeded in time, significantly lower NH_4_^+^ concentrations were first observed at sampling locations closer to the injection well with large increases along column lengths. After injecting 45 L, almost no changes in NH_4_^+^ concentrations were observed near the outlet well (distance of 3.58 m), however, NH_4_^+^ concentrations were reduced to much lower values between 4 mM and 27 mM near the inlet well (distance of 0.15 m) in all columns. When comparing trends in time, spatial NH_4_^+^ concentrations were similar between Columns 1, 2, and 5, however, Column 3 and 4 trends were significantly different. Despite lower pre-treatment NH_4_^+^ concentrations in Column 3 prior to rinsing, trends in time were similar to other Concrete Sand columns when rinse volumes were less than ≈270 L. When additional rinse solution was applied to Column 3, however, continued reductions in NH_4_^+^ were not observed and most locations had concentrations exceeding 1 mM. Reduced NH_4_^+^ removal in Column 3 may have resulted from limited urea hydrolysis during the treatment period, the presence of more unoccupied cation exchange sites, and thus increased retardation of NH_4_^+^ transport. Additionally, lower pH values observed in this column during rinsing, may have resulted in more ammonium existing in the charged form NH_4_^+^ rather than NH_3_. In Column 4, greater NH_4_^+^ removal was again observed for similar injection volumes, despite having a larger column pore volume. Higher exchangeable cation contents measured in Delta Sand may have prevented NH_4_^+^ from interacting with soil surfaces during rinsing, thus improving NH_4_^+^ removal efficiency. After injecting 525 L of rinse solutions, NH_4_^+^ concentrations were below 15 mM, 6 mM, 19 mM, 0.4 mM, and 4 mM at all locations in Columns 1 through 5, respectively. Following the 12 hour stop flow period, however, NH_4_^+^ concentrations generally increased and gradients in concentrations across columns became less pronounced likely due to equilibration of solutions with sorbed NH_4_^+^ concentrations and diffusion. Figure 6 presents changes in NH_4_^+^ concentrations during the stop flow period versus column length. Most locations had NH_4_^+^ increases between 1 and 5 mM during the stop flow period. Again, Column 1, 2, and 5 showed similar trends, with Column 3 and 4 differing. In Column 3, much smaller increases in NH_4_^+^ concentrations were observed with a single location achieving a 6.1 mM reduction during the retention period. Similarly, in Column 4, increases in concentrations were much lower than other columns and were generally near 0.3 mM. Smaller increases in NH_4_^+^ concentrations in these columns during the stop flow period may reflect more limited desorption of NH_4_^+^ resulting from less NH_4_^+^ exposure during treatments (Column 3) and saturation of sorption sites and limited NH_4_^+^ and soil interactions (Column 4).

**Figure 7:**
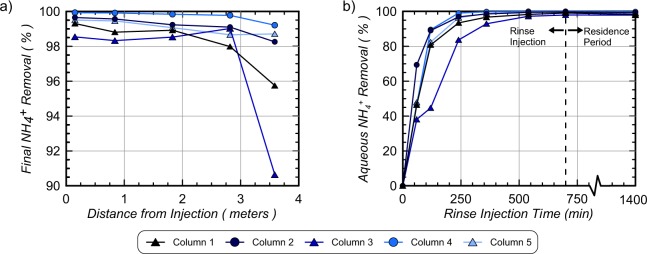
(**a**) Final NH_4_^+^ removal following the 12-hour stop flow period (in percent) versus column length and (**b**) cumulative NH_4_^+^ removal (in percent) versus rinse injection time.

**Figure 8:**
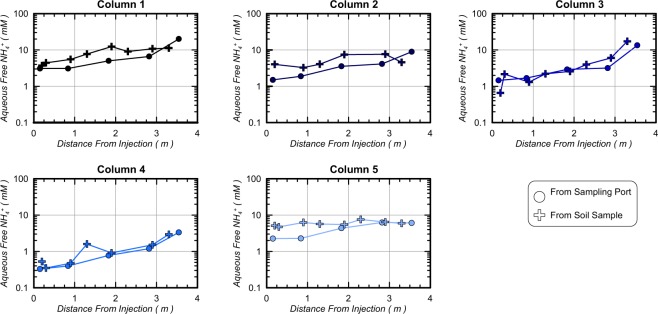
Comparison of aqueous NH_4_^+^ concentrations between solution samples obtained from aqueous sampling ports (after the 12 hour stop flow period) and physical soil samples obtained post-rinsing.

Figure 7 presents (a) removal of pre-rinsing NH_4_^+^ concentrations (in percent) along column lengths and (b) cumulative NH_4_^+^ removal (in percent) for all columns in time. As shown in Fig. 7a, all locations achieved greater than 95.7% NH_4_^+^ removal following rinsing and the 12-hour residence period, with the exception of the most distal location in Column 3 (90.6% removal). At distances less than 2.82 m, greater removal was observed with all columns achieving greater than 98.0% NH_4_^+^ removal. Column 4 achieved the highest NH_4_^+^ removal of all columns with all locations achieving values above 99.2% removal. In order to better understand temporal changes in NH_4_^+^ removal, spatial contours of NH_4_^+^ at various times (Fig. 5) were integrated along column lengths to estimate cumulative NH_4_^+^ removal in time. As shown in Fig. 7b, all columns started with 0% removal before rinsing, and achieved between 38% and 69% NH_4_^+^ removal after injecting only 45 L (60 min). With increased rinse injection volumes, columns exhibited similar removal trends with the exception of Column 3, which achieved less removal in time. In all enriched columns, greater than 80% removal was achieved after injecting 90 L (120 min) and greater than 93% removal was achieved after injecting 180 L (240 min). Column 3, however, required near 270 L (360 min) to obtain 93% removal. Immediately following rinse injections, cumulative removal was 99.1%, 99.7%, 97.9%, 99.9%, and 99.6%, in Columns 1 through 5, respectively. However, after the stop flow period, removal percentages decreased by up to 0.9% and were 98.2%, 99.2%, 97.9%, 99.8%, and 99.1%, in Columns 1 through 5, respectively. Figure 8 presents NH_4_^+^ concentrations measured in aqueous solutions obtained from sampling ports after the stop flow period as well as solutions extracted from moist soil samples obtained post-rinsing at various locations. As shown, values obtained from both methods resulted in similar magnitudes and trends in NH_4_^+^ concentrations along columns suggesting that aqueous samples obtained from sampling ports were generally representative of internal soil conditions. NH_4_^+^ concentrations from soil samples, however, were consistently slightly higher than sampling port values suggesting that greater concentrations of NH_4_^+^ may have resided more locally around soil particle surfaces. Despite these differences, soil sample trends indicated post-rinsing NH_4_^+^ concentrations between 3.7 mM and 17 mM in Column 1, 2, 3, and 5 with greater removal and values between 0.3 mM and 2.9 mM in Column 4.

**Figure 9:**
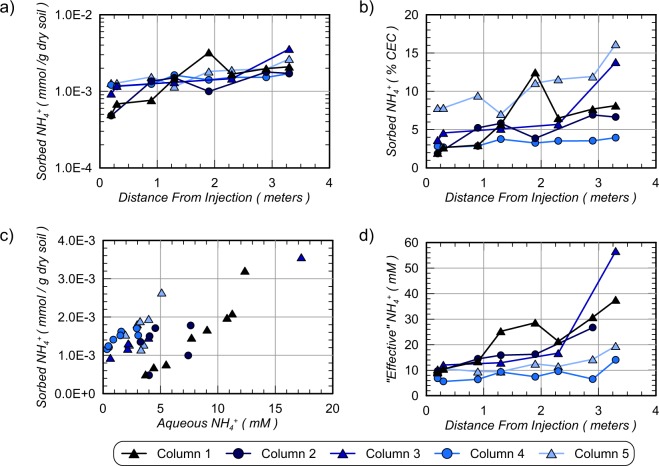
(**a**) Sorbed NH_4_^+^ masses versus column lengths, (**b**) sorbed NH_4_^+^ masses as a percentage of soil CEC versus column lengths, (**c**) sorbed NH_4_^+^ masses versus aqueous NH_4_^+^ concentrations, and (d) “effective” aqueous NH_4_^+^ concentrations versus column lengths.

Figure 9a presents measurements of sorbed NH_4_^+^ masses as determined from KCl extracted soil versus free solution NH_4_^+^ measurements (Fig. 8). As shown, columns had measurable sorbed NH_4_^+^ masses that varied between 4.8 × 10^−4^ and 3.6 × 10^−3^ millimoles of NH_4_^+^ per gram of dry soil. In all columns, sorbed NH_4_^+^ masses increased with distance from the injection well location, suggesting reduced NH_4_^+^ removal from soil surfaces at larger distances. Figure 9b presents similar sorbed NH_4_^+^ masses as a percentage of soil cation exchange capacities. As shown, all sorbed NH_4_^+^ masses were between 1.9% and 16.1% of the total CEC of soil materials. Large differences were observed between soil types with Concrete Sand columns (Column 1, 2, 3) achieving average sorbed NH_4_^+^ masses of 5.8% CEC, Covelo Sand (Column 5) achieving the highest average sorbed NH_4_^+^ masses of 10.3% CEC, and Delta Sand (Column 4) achieving the lowest average sorbed NH_4_^+^ masses of 3.3% CEC. Figure 9c presents measurements of sorbed NH_4_^+^ masses with corresponding aqueous NH_4_^+^ concentrations obtained from soil samples. As expected, for all soil materials, when aqueous NH_4_^+^ concentrations were higher, sorbed NH_4_^+^ masses also increased. The apparent linear relationship between sorbed NH_4_^+^ masses and aqueous NH_4_^+^ concentrations in equilibrium with soil surfaces suggested that NH_4_^+^ sorption in columns may be reasonably described by a Freundlich adsorption isotherm over the concentrations observed^41^. Linear relationships between free NH_4_^+^ concentrations and sorbed NH_4_^+^ masses have been similarly observed in other studies examining ammonium sorption kinetics in soils^42,43^. Furthermore, this correlation suggests that sorbed NH_4_^+^ concentrations were likely higher prior to rinsing and were reduced during the rinsing process. This is consistent with the higher removed NH_4_^+^ concentrations estimated from outlet well measurements (Fig. 4). In order to better understand the amount of NH_4_^+^ remaining on soil particle surfaces, “effective” aqueous NH_4_^+^ concentrations were calculated assuming that all sorbed NH_4_^+^ was instead available to surrounding aqueous solutions. Figure 9d presents “effective” NH_4_^+^ concentrations with distance along all columns computed from known column pore volumes and soil masses. As shown, effective NH_4_^+^ concentrations ranged between 5.6 mM and 56.6 mM for all columns. This suggested that if sorbed NH_4_^+^ masses entered into free solution, aqueous NH_4_^+^ concentrations would increase by 0.05 mM to 42.8 mM. Again, the highest effective NH_4_^+^ concentrations were calculated for Column 3 and the lowest effective NH_4_^+^ concentrations were in Column 4. While these NH_4_^+^ ions remained sorbed to soils under conditions present during the stop flow period, sorbed NH_4_^+^ may present challenges related to desorption over time as groundwater solutions are transported through treated locations.

**Figure 10:**
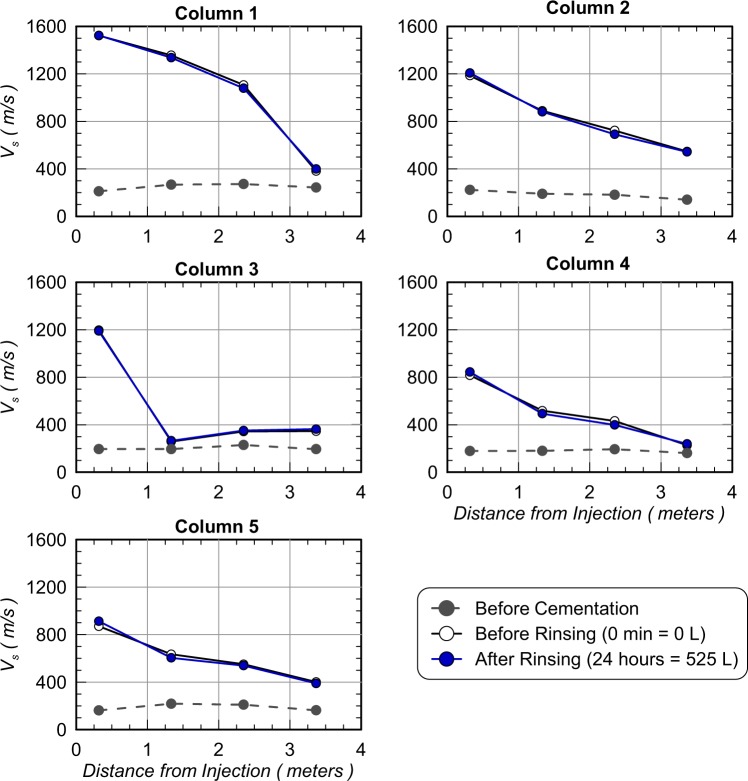
Soil shear wave velocities (V_s_) versus column lengths for all columns before cementation, after cementation and immediately before rinsing, and after rinsing.

**Figure 11:**
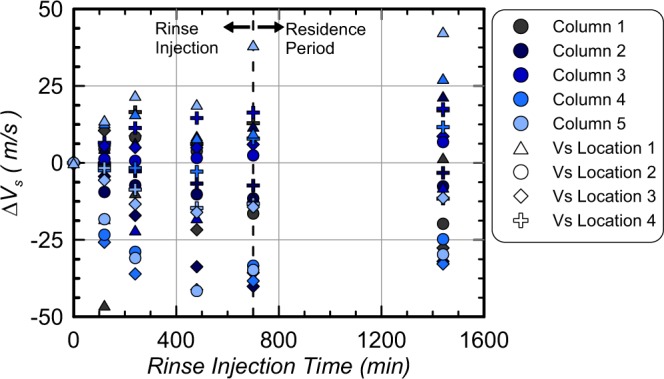
Changes in soil shear wave velocities (V_s_) for all bender element sensor locations in all columns versus time since the start of rinsing injections.

Figure 10 presents contours of soil V_s_ measured along columns lengths for all columns before cementation injections, after cementation but before rinsing, and after rinsing injections following the stop flow period. All columns had similar initial V_s_ values, however, after cementation treatments, large differences in V_s_ distributions were observed between columns and were reflective of differences in bio-cementation distributions. In Column 1, which had the highest ureolytic activity, high magnitudes of cementation were observed at distances less than 2.35 m with V_s_ values ranging from 1107 m/s to 1522 m/s with a large reduction in V_s_ to 382 m/s at a distance of 3.37 m. In Column 2, which contained the same sand but had a lower ureolytic activity, V_s_ values ranged between 723 m/s and 1186 m/s at distances less than 2.35 m, however, a V_s_ of 546 m/s was obtained at 3.37 m, which was significantly greater than Column 1. In Column 3, which was augmented, a V_s_ of 1197 m/s was obtained near the injection well, however, little cementation was detected at distances greater than 0.31 m. In Column 4 and 5, which had lower ureolytic activity, similar trends were observed along column lengths as Column 2, with slightly lower V_s_ values between 817 m/s and 871 m/s measured at a distance of 0.31 m and values between 227 m/s and 401 m/s measured at a distance of 3.37 m. For all columns, V_s_ values before and after rinsing differed by no more than ± 50 m/s suggesting that little dissolution or precipitation had occurred. To further examine trends during rinsing, changes in V_s_ values were plotted in time during rinse injections for all columns and bender element locations (Fig. 11). No consistent trends between columns or measurement locations were observed. Immediately after the start of injections, locations had changes in V_s_ values between −46 m/s and +14 m/s, however, following the stop flow period changes between −33 m/s and +43 m/s were observed. These V_s_ results suggest that rinse injections likely only resulted in minor changes in calcite contents between −0.2% and +0.3% by mass following previous relationships established by Gomez and DeJong (2017)^7^.

## Conclusions

The production of NH_4_^+^ by-products during ureolytic bio-cementation presents a significant challenge for the technology, which must be addressed if MICP is to attain widespread acceptance as an environmentally-conscious ground improvement alternative. In this study, five 3.7-meter long soil columns containing three different sandy soils were treated using different bio-cementation treatment techniques to investigate NH_4_^+^ by-product removal following ureolytic bio-cementation. During treatments, differences in enriched and augmented ureolytic activity were achieved and resulted in differences in bio-cementation distributions. While all enriched columns achieved near full hydrolysis of applied 250 mM urea injections over 24 hours (Column 1) and 48 hours (Column 2, 4, 5), the augmented Column 3 achieved limited urea hydrolysis and less NH_4_^+^ production during cementation treatments. The localization of ureolytic activity and cementation observed near the injection well in Column 3 was attributed to the filtration of *S. pasteurii* bacterial cells during augmentation. Following cementation, a single 525 L volume of a high pH and high ionic strength rinse solution (200 mM CaCl_2_, pH ≈10.0) was applied to each column and NH_4_^+^ removal and cementation integrity were monitored. NH_4_^+^ concentrations observed at the outlet well were compared to expected trends for a passive tracer and results suggest that NH_4_^+^ transport was retarded by soil-ion interactions with significantly greater NH_4_^+^ removal than expected due to removal of sorbed NH_4_^+^ from soil surfaces. When spatial changes in NH_4_^+^ concentrations were examined during rinsing, large gradients in NH_4_^+^ concentrations were observed across columns, however, NH_4_^+^ concentrations below 19 mM were observed at all locations immediately after rinsing. After a 12-hour stop flow period, increases in NH_4_^+^ concentrations between 1 and 5 mM at most locations were observed, with final cumulative NH_4_^+^ removal between 97.9% and 99.8%, achieved for all columns. Greater NH_4_^+^ removal observed in Column 4 was attributed to higher concentrations of exchangeable cations present in the marine soil, which may have limited interactions between NH_4_^+^ and soil minerals. In contrast, limited NH_4_^+^ removal observed in Column 3 was believed to have resulted from both the presence of more unoccupied sorption sites from limited urea hydrolysis and lower pH values observed during rinsing. Following KCl extraction of post-rinsing soil samples, measurements suggested that significant NH_4_^+^ remained sorbed to soil surfaces, which may present challenges related to NH_4_^+^ desorption over time. Finally, soil V_s_ measurements suggested that NH_4_^+^ removal had no significant effect on cementation integrity. While these results suggest that NH_4_^+^ can be successfully removed from aqueous solutions residing in bio-cemented soils, acceptable limits for aqueous NH_4_^+^ concentrations and sorbed NH_4_^+^ masses will likely be governed by site-specific requirements and may require further investigation of rinsing and management techniques.

## Data Availability

The datasets generated during and/or analyzed during the current study are available from the corresponding author on reasonable request. All measured data presented in the figures of this paper will be available through the NSF DesignSafe-CI Data Depot repository (https://www.designsafe-ci.org/data/browser/public/) under project number PRJ-2467.
